# A novel androgen-regulated isoform of the TSC2 tumour suppressor gene increases cell proliferation

**DOI:** 10.18632/oncotarget.1405

**Published:** 2013-10-21

**Authors:** Jennifer Munkley, Prabhakar Rajan, Nicholas P. Laferty, Caroline Dalgliesh, Robert M. Jackson, Craig N. Robson, Hing Y. Leung, David J. Elliott

**Affiliations:** ^1^ Institute of Genetic Medicine, Newcastle University, Newcastle-upon-Tyne, United Kingdom,; ^2^ Beatson Institute for Cancer Research, Glasgow, United Kingdom,; ^3^ Institute of Cancer Sciences, University of Glasgow, Glasgow, United Kingdom,; ^4^ Northern Institute for Cancer Research, Newcastle University, Newcastle-upon-Tyne, United Kingdom.

**Keywords:** TSC2, mTOR, mRNA isoform, androgen, prostate cancer

## Abstract

*TSC2* (*Tuberous sclerosis complex 2*) is an important tumour suppressor gene, mutations within which are linked to the development of tuberous sclerosis and implicated in multiple tumour types. TSC2 protein complexes with TSC1 and blocks the ability of the Rheb (Ras homolog enriched in brain) GTPase to activate mTOR (mammalian target of rapamycin), a crucial signal transducer which regulates protein synthesis and cell growth. Here, we report the characterisation of a novel isoform of TSC2 which is under direct control of the ligand-activated androgen receptor. TSC2 isoform A (*TSC2A*) is derived from an internal androgen-regulated alternative promoter and encodes a 508-amino acid cytoplasmic protein corresponding to the C-terminal region of full-length TSC2, lacking the interaction domain for TSC1 and containing an incomplete interaction domain required for Rheb inactivation. Expression of TSC2A is induced in response to androgens and full-length TSC2 is co-ordinately down-regulated, indicating an androgen-driven switch in TSC2 protein isoforms. In contrast to the well-characterised suppressive efect on cell proliferation of full-length TSC2 protein, both LNCaP and HEK293 cells over-expressing TSC2 isoform A proliferate more rapidly (measured by MTT assays) and have increased levels of cells in S-phase (measured by both Edu staining and FACS analysis). Our work indicates, for the first time, a novel role for this well-known tumour suppressor gene, which encodes an activator of cell proliferation in response to androgen stimulation.

## INTRODUCTION

Androgens drive prostate growth and the development of prostate cancer (PCa), the most common male cancer, via the cognate androgen receptor (AR), which activates a number of known molecular switches [1-4]. The AR exerts its transcriptional effects by binding to DNA sequences termed androgen response elements (AREs) associated with androgen-regulated genes. A number of androgen-regulated genes and pathways have been identifed in PCa, including cell cycle regulators, and biosynthetic, glucose uptake and glycolysis pathways, and the master regulators thereof such as calcium/calmodulin-dependent protein kinase 2 (CAMKK2) [4].

Using a transcriptome-wide approach, we recently demonstrated that androgens can also regulate expression of alternative mRNA isoforms [5], which might also mediate the cellular response to androgens, and have roles in clinical PCa. Alternative mRNA isoforms can have distinct functions in the cell, and there is emerging evidence to suggest that expression of specific splice isoforms derived from PCa-relevant genes, including the AR itself [6], can contribute to PCa biology [7]. We previously identified a novel and previously uncharacterised mRNA isoform made from *TSC2* (*Tuberous Sclerosis Complex 2*) which is a direct and rapidly-activated target of the AR [5].

The *TSC2* gene was initially identified in the development of tuberous sclerosis, a systemic disorder characterised by the development of benign hamartomas [8]. TSC2 protein forms a heterodimeric tumour suppressor complex with TSC1, which is at the crossroads of many different signalling pathways, and serves as a nexus for integrating extracellular growth factor signalling and nutritional availability [9, 10]. The TSC2 protein contains a GTPase-activating protein (GAP) domain and acts as a GTPase inactivating protein for Rheb (Ras homolog enriched in brain) [11]. Rheb acts downstream of TSC2 to activate mTOR (mammalian target of rapamycin) a crucial signal transducer which regulates protein synthesis and cell growth [12-14].

Since *TSC2* is a direct and rapid target of the AR in PCa cells, we aimed to further characterise this novel *TSC2* transcription unit in PCa.

## RESULTS

### A novel isoform of TSC2 protein is induced in response to androgens

**Figure 1:**
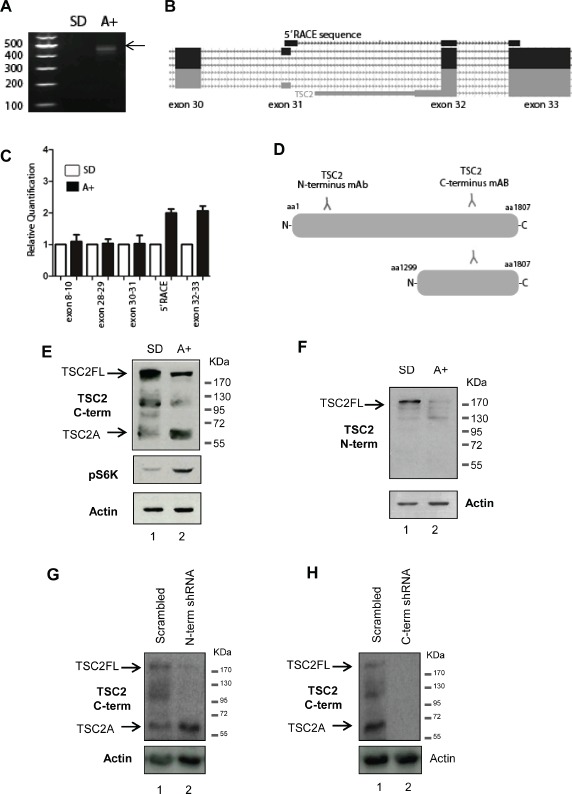
Identification of a novel androgen regulated protein isoform encoded by the tumour suppressor gene *TSC2* in PCa cells (A) A novel internal initiation site from the *TSC2* gene was identified in 24-hour androgen stimulated LNCaP cells using 5′ RACE. First strand cDNA synthesis was primed using a gene-specific primer to *TSC2* exon 34 and a product was obtained for LNCaP cells treated with the synthetic androgen R1881 (A+), which was absent from steroid-depleted (SD) cells. (B) Visualisation of the sequenced 5′ RACE product on the UCSC genome browser indicated the androgen regulated *TSC2* isoform contained a novel 5′ UTR, with a start codon at the beginning of exon 32. (C) Real-time qPCR using primer pairs to specific exons of *TSC2* confirmed the location of the androgen-indued TSC2 internal 5' end. (D) Detection of TSC2 isoforms in LNCaP cells using antibodies specific to the N- and C terminus of the protein revealed an androgen inducible band, named isoform A, of approximately 60kDa detectable using the C-terminal antibody (E) which was absent when the same samples were probed with the N-terminal antibody (F). Anti-p70-S6K was used as a control to confirm the response to androgens. Notably, as well as induction of TSC2A, there was also a reduction in full-length TSC2 protein levels following androgen treatment detectable by both TSC2 antibodies. Stable transfection of LNCaP cells with shRNA targeting full-length TSC2 mRNA but not TSC2A (labelled N-term shRNA) showed that although there is loss of the full-length protein, TSC2A is still present, indicating it is not a degradation product of the full-length protein (G). Stable transfection with shRNA targeting both isoforms (labelled C-term shRNA) resulted in the loss of both proteins, demonstrating the specificity of the C-terminal TSC2 antibody (H).

To precisely identify the *TSC2* transcriptional initiation site associated with androgen stimulation, we first carried out 5′ RACE using previously extensively verified RNA [5] derived from both steroid-deplete and androgen-treated LNCaP cells. We identified a novel androgen-regulated 5′ RACE product absent from steroid-deplete cells. Sequence analysis of this 5′ RACE product indicated a transcript initiating upstream of *TSC2* exon 32. We confirmed the location of this androgen-regulated 5′ end using RT-qPCR to monitor upstream and downstream transcription levels in both steroid-deplete and androgen-treated LNCaP cells. The novel androgen-regulated mRNA isoform (which we termed TSC2 isoform A) contained a 5′ UTR upstream of a translational start codon at the beginning of exon 32, and was predicted to encode a 56 kDa, 508 amino acid protein (Figure 1A,B,C).

To test if *TSC2A* mRNA isoform is translated into protein we performed western blot analyses of protein extracted from LNCaP cells grown in androgen-deplete and androgen-stimulated cells. Antibodies specific to the C-terminus of TSC2 protein detected a shorter protein isoform of approximately the predicted size expected from translation of *TSC2A* (~56kDa), in addition to the expected full-length TSC2 protein (~200 kDa) in androgen treated cells. Corresponding to the C-terminal location of TSC2A in the full-length protein, this 56 kDa species was not detected by an antisera specific to the N-terminus of full-length TSC2 (Figure 1D,E,F). This result was observed in at least six independent experimental repeats. In addition to the androgen-regulated induction of TSC2A expression in LNCaP cells, we also observed a reduction in expression of full-length TSC2 protein as reported previously [15].

In order to confrm the specificity of the band corresponding to TSC2A we stably transfected LNCaP cells grown in full media with shRNAs targeting the full-length TSC2 mRNA, but not the TSC2A isoform. The results showed clearly that despite the loss of full-length TSC2, a band corresponding to the TSC2A protein is still detected by the C-terminal antibody (Figure 1G). This band is absent when shRNA targeting both isoforms is used (Figure 1H).

The specificity of the antibodies was confirmed further by detection of recombinant TSC2A in HEK293 cells by the C-terminal but not the N-terminal antibody (Supplementary Figure A). We also cloned the open reading frame (ORF) of *TSC2A* into an expression vector, and used this to make stable LNCaP cell lines. Increased levels of TSC2A were detected in the stably expressing cell line compared with the control cell line (made with empty vector). The protein encoded by the ORF of TSC2A exactly co-migrated with the endogenous TSC2A protein in the control cell line, thus confirming the expected size of this protein in LNCaP cells (Supplementary Figure B).

### TSC2 isoform A expression in PCa *in vitro* and *in vivo*

**Figure 2:**
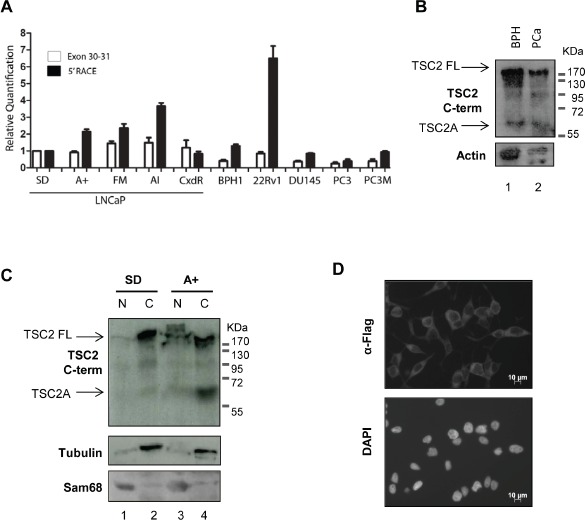
Expression of TSC2 isoform A is detected in clinical PCa samples and is a cytoplasmic protein (A) Real-time qPCR using primer pairs to exons 30-31 and to the start of the 5'RACE product identified in Figure 1 were used to interigate the presence of TSC2A in a variety of PCa cell lines. An increase in the 5'RACE product relative to exons 30-31 in a number of cell lines was observed indicating detection of TSC2A transcript. (B) Both full-length and endogenous TSC2 isoform A were detected by western blotting in two clinical prostate tumour samples using the C-terminal antibody. (C) LNCaP cells were fractionated into nucleus (N) and cytoplasm (C) and analysed by SDS-PAGE and western blotting. Both endogenous TSC2 isoform A and full-length TSC2 were detected with the TSC2 C-terminal antibody within the cytoplasmic fraction. Sam68 and β-tubulin were used as markers of the nucleus and cytoplasm respectively. (D) Flag-tagged TSC2A protein was ectopically expressed in LNCaP cells and detected in the cytoplasm by indirect immunofluorescence. DNA was detected by counterstaining with DAPI.

We analysed a panel of PCa cell lines using primers just upstream of the start of TSC2A (exon 30-31) and specific to the start of TSC2A (5'RACE), to determine the presence of TSC2A transcript. Expression of our 5'RACE product relative to exons 30-31 was increased in the majority of PCa cell lines studied, indicating that expression of this novel mRNA isoform is not specific to LNCaP cells (Figure 2A). We next carried out western analysis of TSC2 protein expression in a panel of PCa tissues to determine if TSC2A protein is expressed within clinical prostate tumours. Using the C-terminal antibody, we detected a protein corresponding in size to full length TSC2 in two out of the six samples studied, both of which also expressed TSC2A (Figure 2B).

TSC2 protein has been previously shown to localise to the cytoplasm, but can localise to the nucleus under some circumstances [16-19]. To determine the intracellular location of endogenous TSC2A protein, we performed sub-cellular fractionation of LNCaP cells, and analysed nuclear and cytoplasmic fractions by western blotting with the anti-TSC2 (C-terminal specific) antibody. These experiments demonstrated that TSC2A fractionated within the cytoplasmic compartment, similarly to the full-length TSC2 protein (Figure 2C). Furthermore, we performed indirect immunofluorescence using anti-FLAG antibody to specifically study the intracellular localisation of the TSC2A isoform in LNCaP cell lines stably expressing TSC2A fused to the FLAG epitope. TSC2A-FLAG protein localised within the cytoplasm of these stably transfected LNCaP cells (Figure 2D).

### TSC2 isoform A does not inhibit mTOR activation

**Figure 3:**
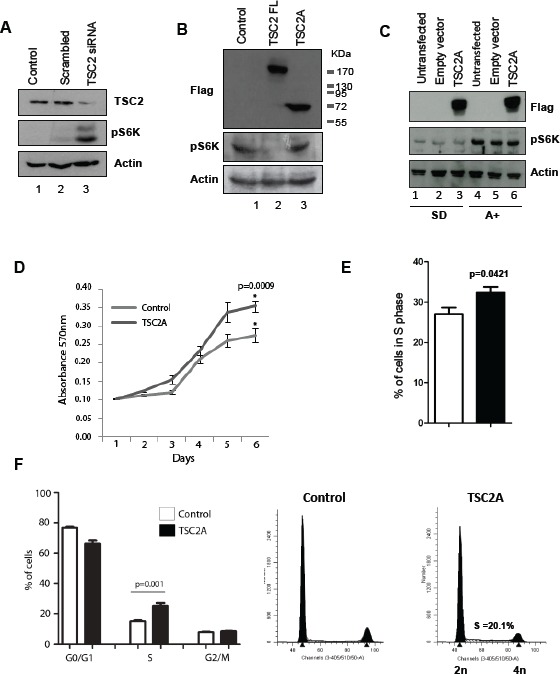
TSC2 isoform A does not inhibit mTOR signalling and increases cell proliferation (A) Knock-down of TSC2 protein by siRNA depletion targeting the C-terminal of the protein causes an increase in mTOR signalling, as measured by changes in phosphorylation of S6K. (B) Conversely, over-expression of full-length TSC2 in HEK293 cells caused a reduction in S6K phosphorylation (lane 2), however unlike full-length TSC2 the TSC2A isoform does not inhibit mTOR activation (lane 3). (C) Over-expression of TSC2A in LNCaP cells did not inhibit mTOR signalling under both steroid-deplete and androgen treated conditions. Analysis of cell proliferation by (D) MTT assay and (E) incorporation of EdU over 6 hours indicated that expression of the TSC2A isoform increases LNCaP cell growth. (F) Similarly, cell cycle analysis of DAPI stained cells by flow cytometry shows that TSC2A decreases the percentage of cells in G1/G0 and increases the percentage of cells in S phase.

Full-length TSC2 protein interacts with TSC1 to inhibit the activation of mTOR by Rheb. When TSC2 protein is lost, (e.g. in tuberous sclerosis), mTOR remains constitutively activated to drive cell growth [20]. To confirm this effect in LNCaP cells, we used siRNA to deplete expression of full-length TSC2. We observed that siRNA-mediated reduction in full-length TSC2 expression was associated with increased mTOR activation, as evidenced by increased phosphorylation levels of S6K, which is downstream of mTOR (Figure 3A, lane 3). Conversely over-expression of full-length TSC2 in HEK293 cells caused a decrease in phosphorylation levels of S6K (Figure 3A, lane 2). In contrast, when TSC2A was over-expressed in these cells, no change in phosphorylation of S6K was observed (Figure 3B, lane 3), indicating that unlike full-length TSC2, TSC2A does not inhibit mTOR activation. We similarly monitored S6K status to test the activity of TSC2A protein expression on activity of the mTOR pathway in LNCaP cells. Although we could detect efficient expression of TSC2A in the stably-transfected cells by western blotting, no inhibition of the mTOR pathway was apparent both in the presence and absence of androgens (Figure 3D, lanes 3 and 6).

### TSC2 isoform A increases cell proliferation

To test if expression of the shorter TSC2A isoform might affect PCa cell proliferation, we monitored cell growth of LNCaP cells either stably transfected with TSC2A or empty vector. In three independent experiments, cells stably expressing TSC2A exhibited a statistically-significant increase in cell proliferation as compared with wild-type LNCaP cells as determined by MTT assays (Figure 3D). To confirm this result, we generated HEK293 cells stably expressing tetracycline-inducible TSC2A. Consistent with the results in LNCaP cells, tetracycline-induced expression of TSC2A increased cell proliferation in HEK293 cells (see Supplementary Figure Ci).

We then monitored incorporation of EdU over a six hour period in LNCaP cells stably-expressing TSC2A. LNCaP cell populations expressing TSC2A exhibited significantly increased proportion of cells in S-phase as compared with wild-type controls (Figure 3E), a result which was confirmed by cell cycle analysis of DAPI stained cells where a corresponding decrease in the proportion of cells in G0/G1 was observed (Figure 3F). Similar results were seen in HEK293 cells expressing TSC2A (Supplementary Figure Cii, iii) confirming that TSC2A increases cell proliferation.

## DISCUSSION

A number of oncogenes and tumour suppressor genes have been shown to have multiple promoters, and the aberrant use of promoters in some genes has been directly correlated to cancer initiation and progression [21, 22]. Technological advances in gene expression profiling and mapping of transcription factor binding are able to identify androgen-dependent changes in promoter usage and alternative isoform expression which normally would not have been detected using standard 3' end arrays [5]. These changes may yield functionally differing protein isoforms which may be important in mediating the androgenic response in prostate development and tumourigenesis.

Here, we report the characterisation of a new isoform of the tumour suppressor gene TSC2 which is derived from an internal androgen-regulated alternative promoter and has potentially oncogenic activity. Androgen stimulation causes a switch in TSC2 protein isoform expression; expression of TSC2A is induced and full-length TSC2 is co-ordinately down-regulated, with overall TSC2 expression levels remaining similar. Dutertre et al. 2010 studied the use of alternative promoters (AP) in response to oestrogens and identified a number of cases where estradiol-regulated a switch in mRNA isoforms, without affecting the overall expression level of each gene [23]. Our findings indicate that AR may be regulating TSC2 isoform expression in a similar way, with significant functional consequences.

Down-regulation or loss of full-length TSC2 has been shown to stimulate cell proliferation [24], whereas over-expression of TSC2 has been shown to have a suppressive role on cell proliferation in a variety of different cell types [25, 26]. In contrast, our results indicate that rather than having a suppressive role on cell growth, expression of TSC2 isoform A actually causes cells to proliferate more rapidly. These data suggest that the mitogenic effect of androgens in PCa cells may be mediated not only by the androgen-dependent reduction in full-length TSC2 expression (and subsequent effects on mTOR signalling), but also by an independent and novel effect mediated specifically by cytoplasmic TSC2A. Our results indicate that the shorter TSC2A protein itself does not act as an inhibitor of mTOR signalling and is consistent with an absent interaction domain for TSC1 and an incomplete Rheb interaction domain [27].

**Figure 4:**
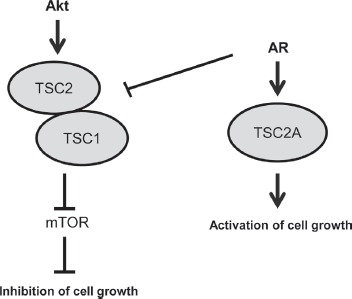
Model: Full-length TSC2 inhibits cell growth, whereas isoform TSC2A activates cell growth In steroid-deplete conditions full-length TSC2 acts in complex with TSC1 to inhibit mTOR activation and have a negative effect on cell growth. In androgen stimulated cells expression of the full-length TSC2 protein is reduced and there is simultaneous induction of isoform TSC2A both of which increase cell proliferation.

The use of alternative promoters within the same gene to encode different mRNA isoforms, with one splice isoform acting as a tumour suppressor and an alternative isoform functioning as an oncogene, has been described previously. The *RASSF1* (*Ras association domain family 1*) gene is a Ras effector which encodes two major mRNA isoforms, *RASSF1A* and *RASSF1C* by alternative promoter selection and alternative splicing [28]. The *RASSF1A* isoform encodes a well-characterised tumour suppressor protein which is often epigenetically silenced in cancer [29, 30], whereas *RASSF1C* which is derived from an alternative promoter has been shown to stimulate proliferation and attenuate apoptosis in breast cancer cells [28]. Our results suggest that full-length TSC2 and TSC2 isoform A may have similar opposing, antagonistic functions within cells with oscillations in expression of TSC2 isoforms influencing PCa cell growth in response to the AR (Figure 4). This may be of clinical significance as TSC2A expression may drive mTOR-independent cell growth, conferring resistance to mTOR inhibition as a therapy for clinical PCa.

## MATERIALS AND METHODS

### Antibodies

The following antibodies were used: anti-TSC2 C-term rabbit polyclonal antibody (4308, Cell Signalling Technology), anti-TSC2 N-term rabbit polyclonal antibody (3635, Cell Signalling Technology), anti-p70-S6K (9234 Cell Signalling Technology), anti-Sam68 rabbit polyclonal antibody (sc333 Santa Cruz Biotechnology), anti-actin rabbit polyclonal antibody (A2668, Sigma), anti-β-Tubulin mouse monoclonal antibody (T5293, Sigma), anti-FLAG mouse monoclonal antibody (F3165, Sigma), normal rabbit IgG (711-035-152 Jackson labs) and normal mouse IgG (715-036-150 Jackson labs).

### DNA Constructs

TSC2A was cloned into pCDNA5-fag (V6520-20, Invitrogen) using BamHI and XhoI, into pCDNA3.1+ (V790-20, Invitrogen) using BamHI and XhoI, and into pX3fag CMV 10 (E7658, Sigma) using EcoRI and BamHI. TSC2 Full length was cloned into pCDNA5-fag using BamHI and BclI.

### Cell Culture

All cells were grown at 37°C in 5% CO_2_. LNCaP cells (CRL-1740, ATCC) were maintained in RPMI-1640 with L-Glutamine (PAA Laboratories, R15-802) supplemented with 10% Fetal Bovine Serum (FBS) (PAA Laboratories, A15-101). Where indicated, medium was supplemented with 10% dextran charcoal stripped FBS (PAA Laboratories, A15-119) to produce a steroid-deplete medium. Cells were then cultured for 72 hours, following which 10nM synthetic androgen analogue methyltrienolone (R1881) (Perkin–Elmer, NLP005005MG) was added (androgen) or absent (steroid-deplete) for the times indicated. Stable LNCaP cell lines were generated by transfecting cells using Lipofectamine 2000 (11668-027, Invitrogen), followed by selection with 300µg/ml Geneticin (Invitrogen, 10131019) (reduced to 150µg/ml following the death of untransfected cells) for at least four weeks. Flp-In™-293 cells (R750-07, Invitrogen) were maintained in DMEM GlutaMax (Invitrogen, 10566-040), supplemented with 10% FBS (PAA Laboratories, A15-101) and stable cell lines generated using the Flp-In T-Rex Core Kit (K6500-01, Invitrogen) according to the manufacturer's instructions. Protein expression was induced using 1 µg/ml tetracycline (T7660, Sigma) for 72 hours.

### Clinical Samples

Full ethical approval was obtained for all human sample collections from the Northumberland, Tyne and Wear NHS Strategic Health Authority Local Research Ethics Committee (Ref: 2003/11).

### 5' Rapid amplification of cDNA ends

The identity of the 5' end of the TSC2 isoform was determined using the 5' RACE system for rapid amplification of cDNA ends (18374-058, Invitrogen) as per the manufacturer's instructions and using the following gene specific primers: exon 33-34 5'-AGGATTGGCTTGTTTGA-3' and exon 32-33 5'-AGGAGACGACTCGCTCGAT-3'. Following amplification, gel bands were purified with the QIAquick Gel Extraction kit (Qiagen, 28704), cloned using a CloneJET PCR Cloning Kit (Fermentas Life Sciences, K1231) and sequenced (Source Bioscience) using a T7 sequencing primer.

### RT-qPCR

Cells were harvested and total RNA extracted using TRIzol (Invitrogen, 15596-026) according to manufacturer's instructions as previously described [31]. RNA was treated with DNase (Ambion) and cDNA was generated by reverse transcription of 1µg of total RNA using the Superscript VILO cDNA synthesis kit (Invitrogen, 11754-050). Quantitative PCR (qPCR) (Applied Biosystems 7900HT) was performed in triplicate on cDNA using SYBR® Green PCR Master Mix (Invitrogen, 4309155). Samples were normalised using the average of three reference genes, GAPDH, β–tubulin and actin. All primer sequences are listed in Supplementary Table 1.

### siRNA

Knockdown of TSC2 was carried out using a pre-designed silencer select siRNA (Ambion S14437) and siPORT NeoFX transfection reagent (AM4510, Invitrogen). Scrambled silencer select siRNA was used as a control (Ambion). ShRNA: TSC2 N-term shRNA target sequence 5'-GAGCCCTCTTCTTTAAGGTCATC-3' was cloned in pKLO.1. TSC2 C-term and scrambled shRNA plasmids were obtained from Addgene (plasmid 15478 and 1864). Stable cell lines were generated by selecting cells with 0.5µg/ml puromycin and expanding single colonies.

### Sub-cellular Fractionation

Protein localisation within the cell was determined by fractionating cells to separate nuclear and cytoplasmic compartments as described previously [32].

### Proliferation assays

MTT cell proliferation assay was carried out as per the manufacturer's instructions (Cayman, 10009365) starting with 20000 cells (LNCaP) or 5,000 cells (HEK293) per well, with 9 replicates per sample. EdU incorporation was measued using the Click-iT® EdU Alexa Fluor® 488 Imaging Kit (Invitrogen, C10337) and counted using ImageJ. Additional cell cycle analysis was carried out using a CyStain® DNA 2 step kit (Partec UK, 05-5005) and FACSCanto II (BD Biosciences). Results were analysed using ModFit under standard settings.

## Supplementary Figure and Table


