# Supplementary material for: A novel androgen-regulated isoform of the TSC2 tumour suppressor gene increases cell proliferation
Source: Oncotarget. 2013 Oct 21;5(1):131–9. doi: 10.18632/oncotarget.1405 (PMC3960195; doi:10.18632/oncotarget.1405)
Supplement: Supplementary file 1 [file oncotarget-05-0131-s001.pdf]

A novel androgen-regulated isoform of the TSC2 tumour suppressor gene increases cell proliferation - Munkley et al

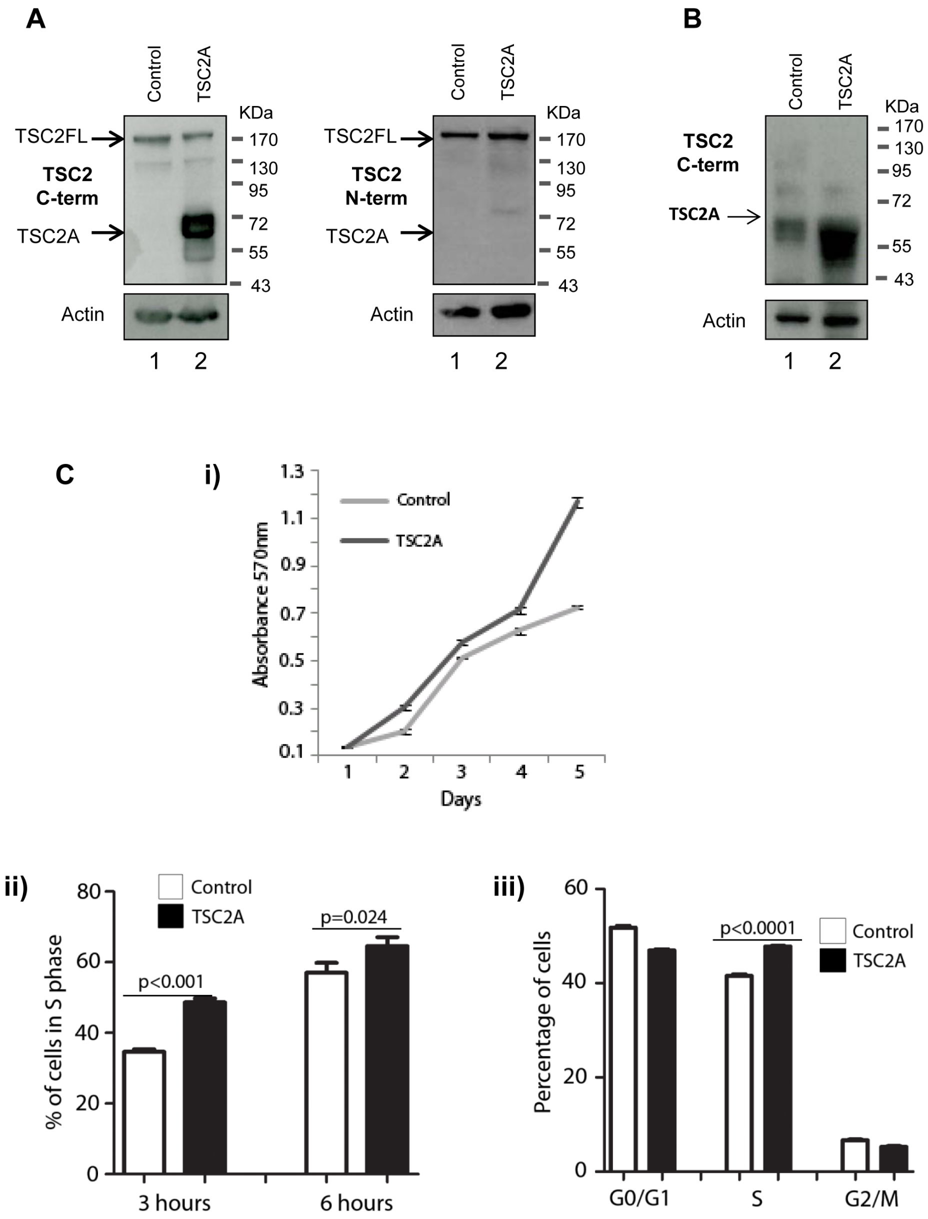

Supplementary data: (A) In HEK293 cells overexpressing TSC2A protein, a protein corresponding in size to TSC2A is detected by the TSC2 C-terminal antibody (i), but not by the N-terminal antibody (ii). (B) The ORF corresponding to use of the *TSC2* internal initiation site was cloned into pCDNA3.1 and LNCaP cells were transfected with this contruct or with a control empty vector to produce stable cell lines. Western blotting of extracts from these stably transfected cells identified a protein of exactly the same size as endogenous TSC2 isoform A. (C) TSC2A increases proliferation of HEK293 cells. Similar to the data shown for LNCaP cells in Figure 2, over-expression of isoform TSC2A also increases proliferation of HEK293 cells.

Supplementary Table 1

	For	Rev
Actin	CATCGAGCACGGCATCGTCA	TAGCACAGCCTGGATAGCAAC
β –tubulin	CTTCGGCCAGATCTTCAGAC	AGAGAGTGGGTCAGCTGGAA
GAPDH	AACAGCGACACCCATCCTC	CATACC AGGAAATGAGCTTGACAA
TSC2 ex8-10	GTTACCCTCTGTCGCACCAT	AAAAACACGGCTCCTCTCAG
TSC2 ex28-29	ATCGGGTCCGTTCCATGT	AGGTTCGTCTTCTCCTGCAC
TSC2 ex30-31	GAGCTGTCTAACGCCCTCAT	TGGACTGGTACAGGGAGGAG
TSC2 5'RACE	GTTCCTGTGCTGGTGGAG	CCTGGCTGGAGACTGAGG
TSC2 ex32-33	GGTTCCTGTGCTGGTGGA	CCTGGCTGGAGACTGAGG
